# Supplementary material for: TGFβ Inhibitor A83-01 Enhances Murine HSPC Expansion for Gene Therapy
Source: Cells. 2023 Jul 31;12(15):1978. doi: 10.3390/cells12151978 (PMC10416825; doi:10.3390/cells12151978)
Supplement: Supplementary file 1 [file cells-12-01978-s001.zip › cells-2521517-supplementary Figures S1-S6.pdf]

Supplementary Information

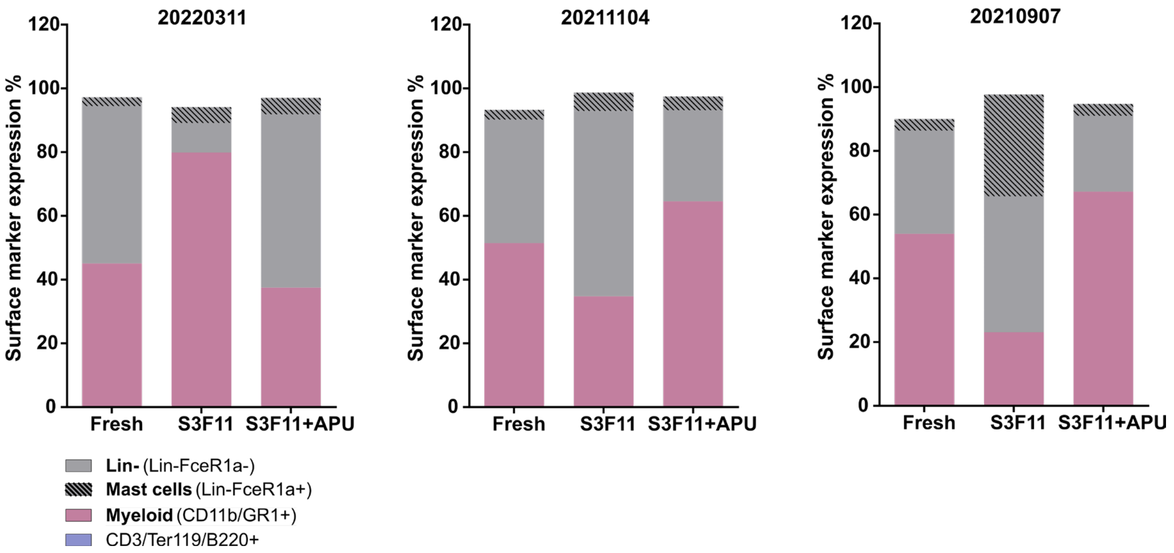

Supplementary Figure S1: Surface marker expression of three individual IVIMs on day -1: IVIMs with the IDs 20220311, 20211104 and 20210907 on the day of transduction (day -1)**.** In every IVIM, the S3F11+APU-Exp cells have a highly comparable immune phenotype like the fresh lin- cells. S3F11-Exp cells are usually ahead of differentiation.

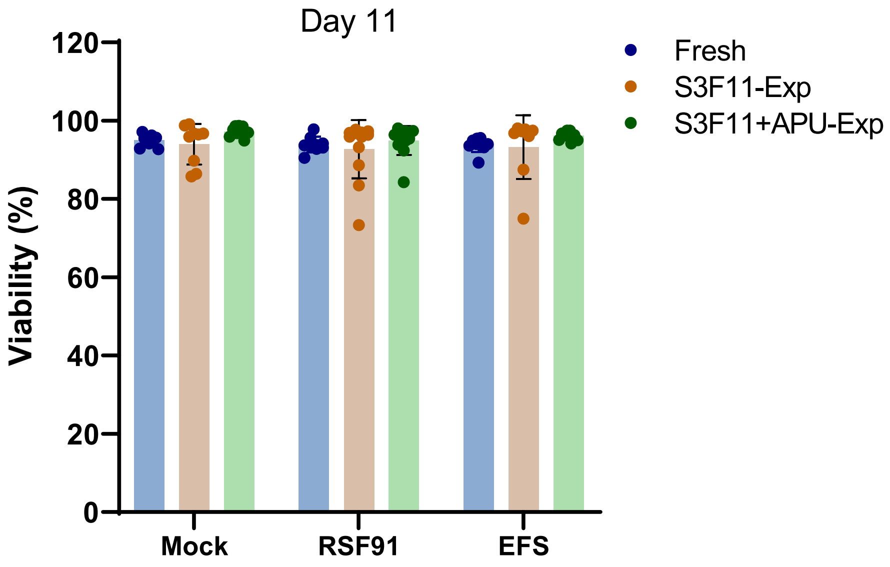

Supplementary Figure S2: Viability of fresh and expanded cells in the IVIM assay on day 11. Exemplary comparison from one time point during n=3 IVIM assays. Fresh and S3F11+APU-Exp cells reached stable viability 11 days after transduction.. Some of the S3F11-Exp samples had lower viability which is in line with their reduced proliferation capacity. No significant differences (p>0.05) were determined by two-way ANOVA analysis.

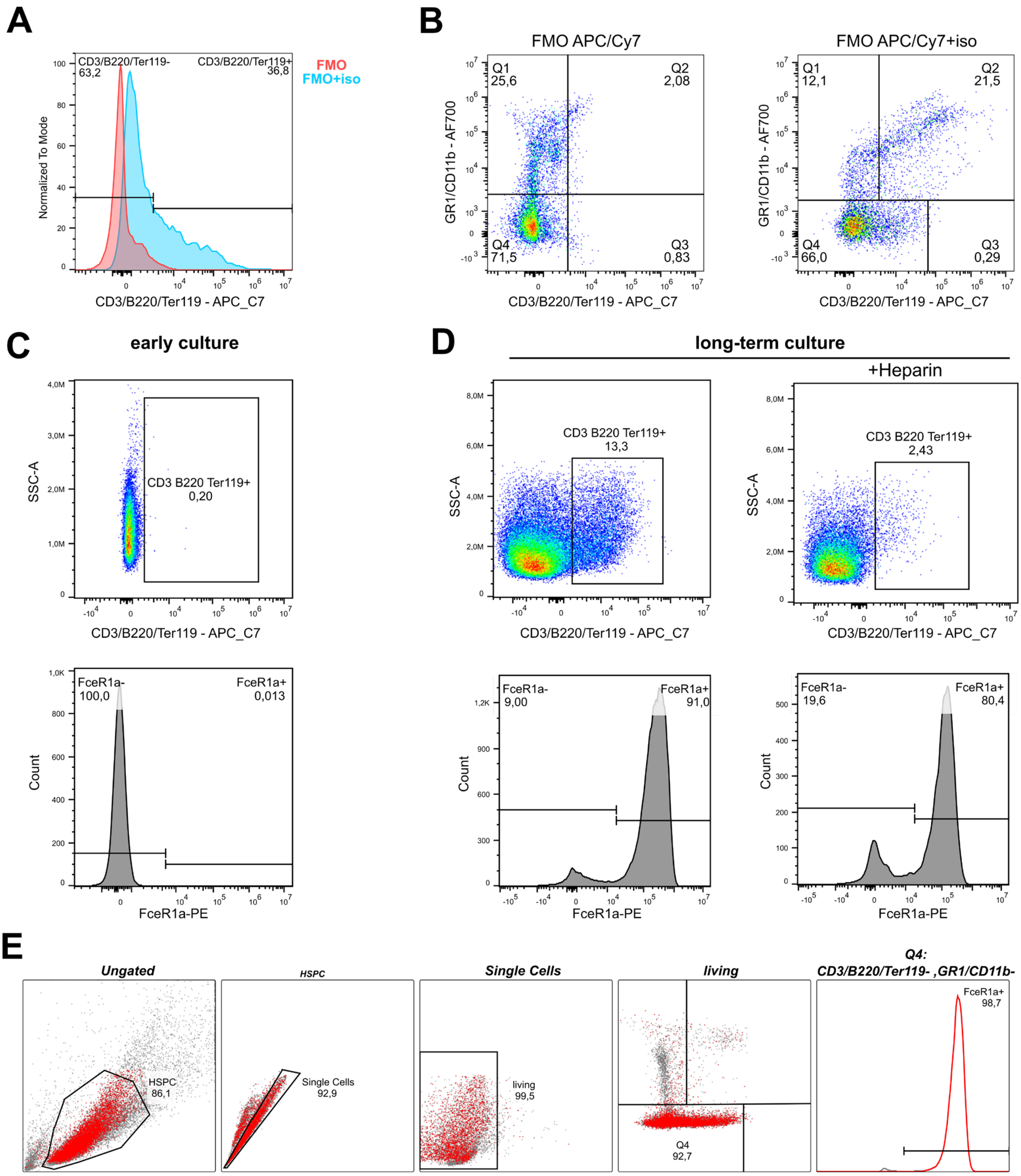

Supplementary Figure S3: Non-specific binding in long-term cultures by mast cells. (A) Isotype control of CD3/B220/Ter119 –APC/Cy7 bound non-specifically and resulted in a dim false-positive population. **(B)** Adjustment of the gating according to the FMO+isotype control. **(C)** Early cultures (exemplary plot from LSK SLAM expansion on day 7) without mast cells did not display non-specific signals. **(D)** Long-term cultures (exemplary plot from an IVIM on day 29, S3F11+APU-Exp mock) consisted mainly of FcεR1α+ mast cells. Pre-incubation with heparin before antibody staining reduced non-specific binding.

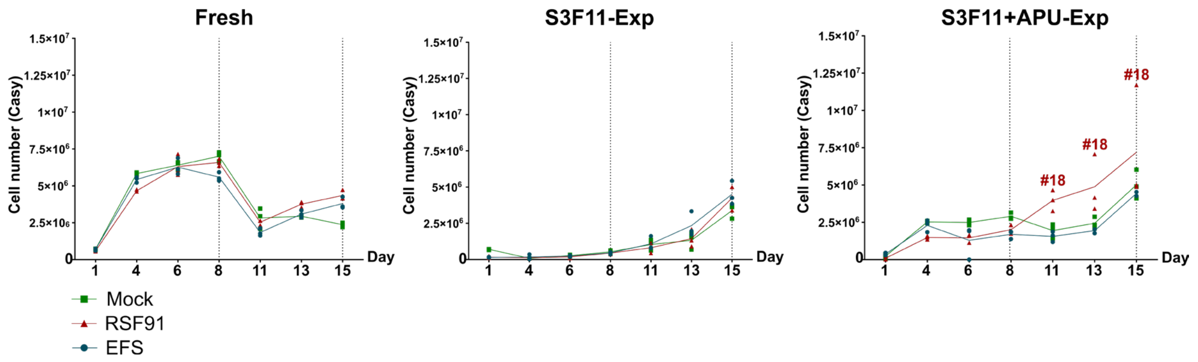

Supplementary Figure S4: Proliferation curves from IVIM 20220311 of fresh, S3F11-Exp and S3F11+APU-Exp cells. Number of viable cells counted with the Casy device during the assay. On day 8, if possible, 1x10[6] cells were seeded and on day 15 cells were replated in low-cell density. Sample#18 in S3F11+APU-Exp is highlighted in red, because of its high proliferation.

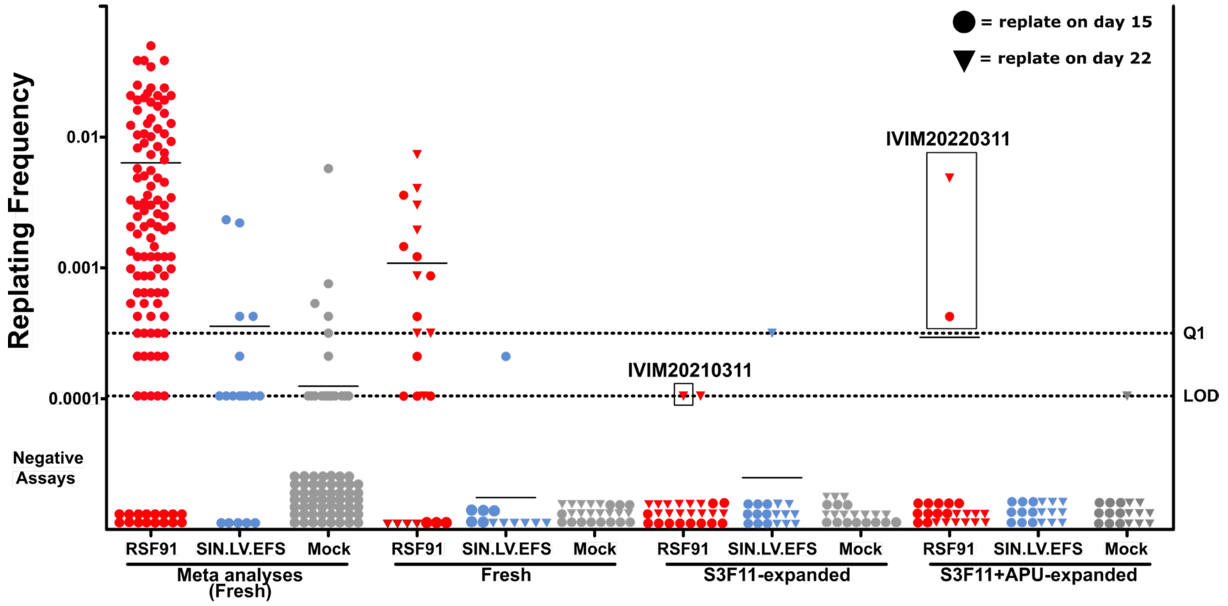

Supplementary Figure S5: Replating frequency (RF) of individual IVIM samples based on the number of positively scored wells after replating either on day 15 (circle) or day 22 (triangle). The threshold for positive mutagenicity is a count of at least three positive wells (Q1 = 0.75 quantile of the RF for RSF91) and the limit of detection (LOD) is equal to one positive well. On the left hand site, the RF of previously performed IVIM assays (meta analyses) are depicted. We compare the RF of the during the study acquired data to meta analyses and see a reduced likelihood for replating in the latest data points. The immortalization events in the two IVIM assays 20210311 and 20220311 are highlighted as these wells gave rise to clones.

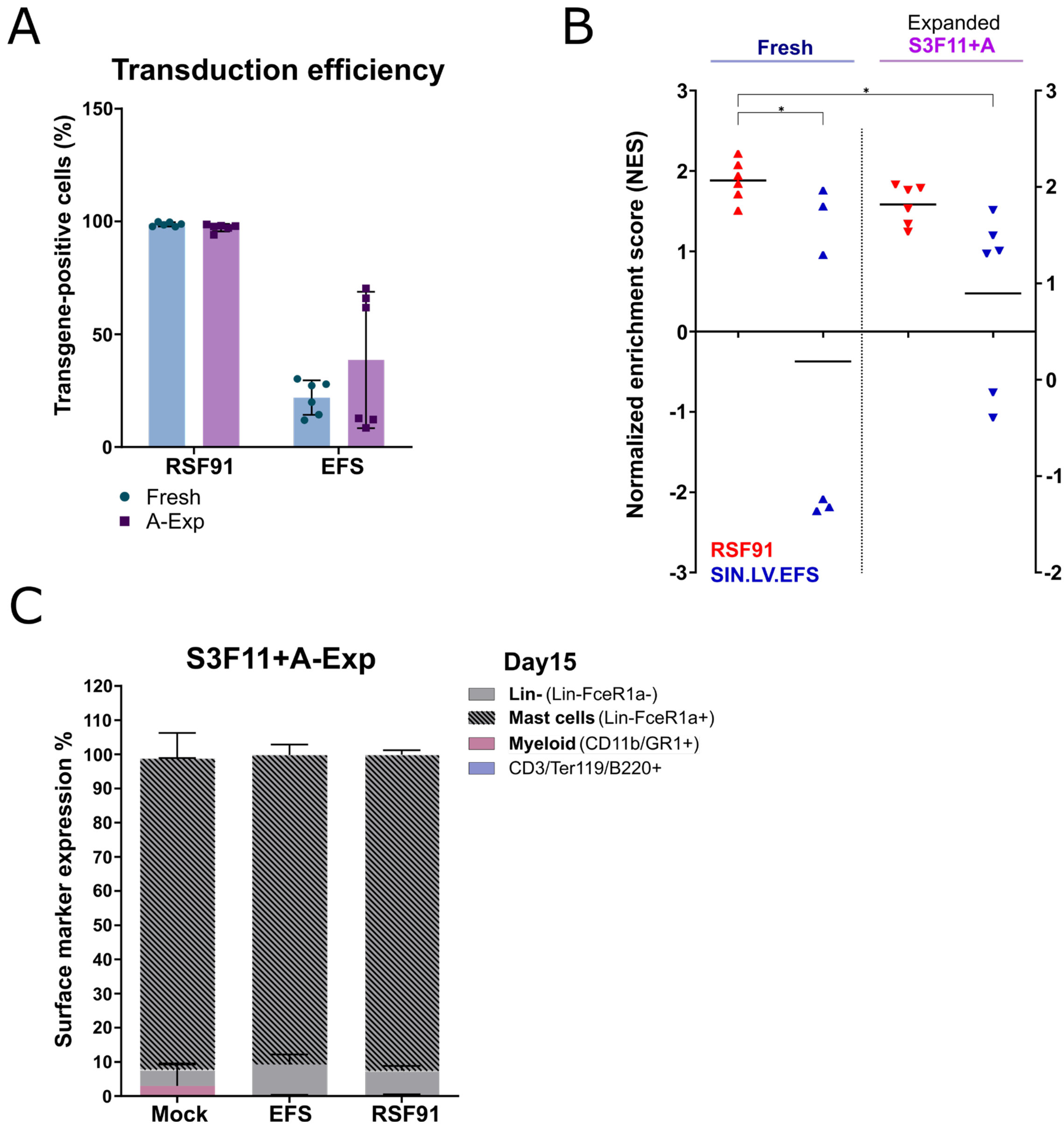

Supplementary Figure S6: IVIM and SAGA using S3F11+A-Exp cells. (A) Percentage of transgene-positive cells on day 15 post-transduction either with RSF91 or SIN.LV-EFS. A-Exp cells and fresh lin- cells were similarly transduced with both vectors. **(B)** NES based on the enrichment in the SAGA gene core set of Fresh and S3F11+A-exp cells. **(C)** Immune phenotype of S3F11+A-Exp cells shows no difference between mock, EFS and RSF91. n=2 IVIMs with technical triplicates. Statistical analysis performed with One-way ANOVA and Kruskal-Wallis' multiple comparisons test, $p<0.05$ = *.
